# MRNA innovation: preparing for arboviruses and the next global outbreak

**DOI:** 10.1097/MS9.0000000000003789

**Published:** 2025-08-28

**Authors:** Fatema Ali Asghar, Maliha Khalid, Muhammad Talha, Aminath Waafira

**Affiliations:** a Karachi Medical and Dental College, Karachi, Pakistan; b Jinnah Sindh Medical University, Karachi, Pakistan; c King Edward Medical University, Lahore, Pakistan; d The Maldives National University, Malé, Maldives

**Keywords:** *Aedes* mosquitoes, arboviruses, mRNA vaccines, outbreak preparedness

## Abstract

Aedes-borne arboviruses, including dengue, Zika, chikungunya, yellow fever, Japanese encephalitis, Rift Valley fever, and Venezuelan equine encephalitis, are rapidly expanding their global reach, with recent outbreaks occurring in non-endemic regions such as southern Europe and the southern United States. Environmental suitability in parts of South America, Asia, and Africa supports continued high transmission potential, while re-emerging pathogens like Oropouche fever further highlight the threat. Current vaccines—though effective in specific contexts—face significant limitations in coverage, safety, and adaptability, underscoring the urgent need for innovative approaches. Advances in mRNA technology, including multivalent self-amplifying RNA platforms, offer a rapid, flexible solution with strong preclinical efficacy against multiple arboviruses. By synthesizing recent epidemiological data, vaccine developments, and translational research, this work calls for accelerated investment, global collaboration, and preparedness strategies to enable timely deployment of next-generation vaccines before the next major outbreak.

*Dear Editor,*

Arboviruses transmitted by *Aedes* mosquitoes are rapidly expanding their global reach, making it essential to identify high-risk regions to better inform public health strategies. *Aedes aegypti* and *Aedes albopictus* mosquitoes have adapted remarkably well to urban environments, with their rapid reproduction and resilience allowing them to thrive across both tropical and temperate climates, complicating control efforts. The geographic range of mosquito-borne diseases such as dengue, chikungunya, Zika, yellow fever, Japanese encephalitis virus (JEV), Rift Valley fever virus (RVFV), and Venezuelan equine encephalitis virus (VEEV) has significantly widened in recent years. Notably, chikungunya and Zika viruses have spread beyond their traditional tropical zones, now appearing in southern Europe and the southern United States. Dengue, in particular, continues to surge, with 2023 and early 2024 marking unprecedented outbreak levels in countries like Brazil, Peru, and Bangladesh^[1]^.

Regions in South America, Asia, and parts of Africa demonstrate high environmental suitability for diseases like dengue, chikungunya, Zika, and yellow fever, indicating favorable conditions for transmission^[2]^. Additionally, the re-emergence of Oropouche fever in Brazil during 2023–2024 further underscores the growing threat posed by arboviral infections in the face of changing ecological and epidemiological landscapes^[3]^. The rising global burden of mosquito-borne arboviruses underscores the urgent need for enhanced data collection, strategic public health planning, and strengthened international cooperation, particularly to accelerate the development of more effective and adaptable mRNA vaccines. Existing research shows that while several vaccines have been approved or are in clinical development, significant limitations remain.

The yellow fever vaccine has long been a cornerstone of disease prevention. In the case of dengue, Sanofi’s Dengvaxia—currently the only FDA-approved vaccine—is restricted to individuals aged 9–16 who have had prior dengue exposure, as it poses a heightened risk of severe illness in seronegative children^[4]^. Takeda’s Qdenga has received approval in select countries, though ongoing evaluation of its safety and efficacy continues. Encouraging results from phase 3 trials of the Butantan-DV vaccine suggest additional potential in dengue prevention.Japanese encephalitis vaccines are already widely administered and highly effective, while vaccine candidates for Rift Valley fever and Venezuelan equine encephalitis are progressing through clinical trials with promising outcomes.

Advances in mRNA technology offer hope for more rapid and flexible vaccine development, especially in the face of emerging outbreaks. Notably, newer candidates like NIAID’s TV003/TV005 have shown strong safety profiles and broad protection. In addition, monovalent and bivalent saRNA-NLC vaccines have demonstrated robust antibody and T cell responses without immune interference and achieved complete protection against lethal Zika and yellow fever infections in animal studies^[5]^. These findings support the continued exploration of multivalent saRNA-based vaccines for broader and more effective protection against arboviral threats.

Given the expanding threat of arboviral diseases and the limitations of current vaccines, the development of next-generation mRNA-based vaccines, particularly multivalent platforms, represents a critical step forward. As recent trials in 2025 begin to assess candidates targeting re-emerging viruses like Oropouche fever, global health systems must prioritize investment, collaboration, and preparedness to ensure rapid deployment of safe, effective vaccines ahead of the next pandemic. Our work is in line with the TITAN Guidelines on the need for transparency in AI use in healthcare^[6]^.

## Data Availability

No data were generated for this manuscript.

## Peer and provenance statement

This article was not commissioned and was externally peer-reviewed.
